# 1750. Evaluation of Antibiotic Allergy in the Ambulatory Setting

**DOI:** 10.1093/ofid/ofac492.1380

**Published:** 2022-12-15

**Authors:** Sarah M Yi, Mary C Barsanti-Sekhar, Amy W Wozniak, Maressa Santarossa, Jenna Adams, Fritzie S Albarillo

**Affiliations:** Loyola University of Chicago Stritch School of Medicine, Forest Park, Illinois; Loyola Medical Center, Park Ridge, Illinois; Loyola University of Chicago Health Sciences Division, Maywood, Illinois; Loyola University Medical Center, Maywood, Illinois; Loyola University Medical Center, Maywood, Illinois; Loyola University Medical Center, Maywood, Illinois

## Abstract

**Background:**

It is not uncommon for patients to have a labeled allergy to antibiotics without thorough investigation of their adverse reactions. The adverse reactions described by some patients are not immune-mediated and these patients are incorrectly labeled as having an antibiotic allergy. As clinical testing for antibiotic allergy is expensive and time intensive, recent initiatives have successfully used patient interviewing to differentiate non-immune mediated reactions from true antibiotic allergies. We investigated the prevalence of patients with non-immune mediated reactions to antibiotics among patients with documented antibiotic allergy by using a standardized questionnaire in the outpatient setting.

**Methods:**

Patients with a documented antibiotic allergy were identified and recruited from 2 clinics located in the greater Chicagoland area. Subjects completed one standardized questionnaire regarding each of their previous adverse reaction to antibiotics. We then evaluated the questionnaire responses to extricate the non-immune-mediated adverse reactions. The full symptom classification can be found in Table 1 and Table 2.

**Figure:**
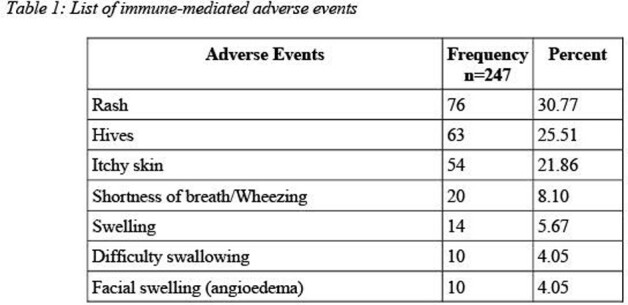

**Figure:**
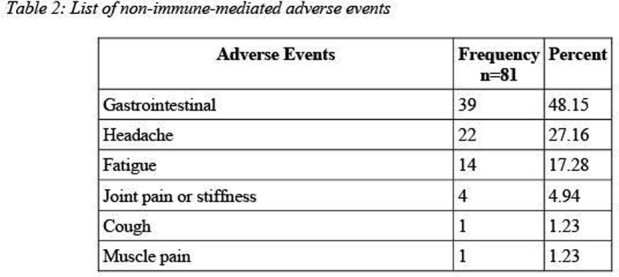

**Results:**

98 patients were recruited with a total of 159 antibiotic questionnaires completed. At the patient level, 18 individuals had no immune-mediated reactions despite antibiotic allergy labels: 18.37% (95% CI: 10.7%, 26.3%). At the antibiotic level, 35 labels had corresponding clinical histories that were likely non-immune mediated: 22.0% (95% CI: 14.7%, 29.4%). Macrolides (8/11, 72.7%), nitrofurantoin (1/2, 50%), and amoxicillin/clavulanate (2/8, 25%) were the antibiotics with the highest percentage of corresponding clinical histories that were likely non-immune mediated (Figure 1). Penicillin was the most prevalently listed antibiotic allergy (43/159, 27.0%), followed by sulfonamides (25/159, 15.7%) and fluoroquinolones (21/159, 13.2%).

**Figure:**
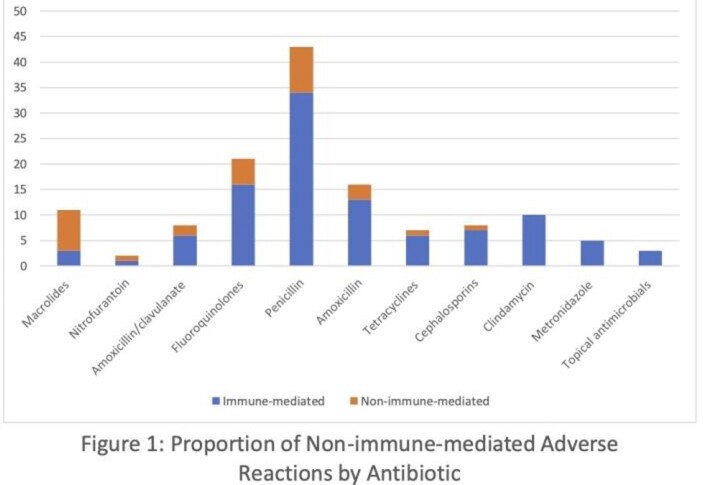

**Conclusion:**

This study demonstrated the feasibility of using a standardized questionnaire to discern true antibiotic allergies from non-immune mediated adverse reactions. The improved accuracy of documented allergies allows for better optimization of antibiotic prescribing.

**Disclosures:**

**All Authors**: No reported disclosures.

